# Magnitude of Neglected Tropical Diseases in Indonesia at Postmillennium Development Goals Era

**DOI:** 10.1155/2016/5716785

**Published:** 2016-04-17

**Authors:** Tri Wibawa, Tri Baskoro Tunggul Satoto

**Affiliations:** ^1^Department of Microbiology, Faculty of Medicine, Universitas Gadjah Mada, Yogyakarta, Indonesia; ^2^Department of Parasitology, Faculty of Medicine, Universitas Gadjah Mada, Yogyakarta, Indonesia

## Abstract

The world will enter the postmillennium development goals 2015 era. The achievements of the millennium development goals (MDGs) as a global development target need to be evaluated. A sustainable new reasonable target is important for neglected tropical diseases (NTD) elimination in Indonesia. This review describes the NTD situation in Indonesia and highlights problems beneath the NTD transmission. Multidisciplinary approach is a promising strategy to help the marginalized people.

## 1. Introduction

The world will enter the postmillennium development goals 2015 era. There have been significant achievements regarding to the millennium development goals (MDGs) as a global development target. The indicators showed reflection of these achievements, such as decreasing global poverty rate, increasing the number of children going to school, decreasing child death rate, increasing access to clean water, and also increasing malaria, HIV/AIDS, and tuberculosis control investment. MDGs were evaluated in the year 2015, and there will be remaining new challenges to sustain the achievement at post-2015 era. Numerous important issues have been raised by United Nation (UN), which are poverty and hunger elimination, improving health and education, sustainable cities, combating climate changes, and also ocean and forest conservation [1].

In MDGs, HIV/AIDS, malaria, and major diseases are clearly mentioned as global development goals. In this case, there will be plenty of diseases that are not a major concern of the world. Questions were raised concerning neglected tropical disease (NTD) which is definitely out of the highlight. Will these diseases be included in the ambitious post-MDGs sustainable development? The new Sustainable Development Goals (SDGs), known also as the Global Goals, had been established after world leaders gathered on 25 September 2015, at the United Nations in New York to adopt the 2030 Agenda for Sustainable Development and the broader and further agenda than the MDGs.

MDG explicitly stated that HIV/AIDS, malaria, and tuberculosis will be efficiently controlled at the end of the year 2015. The big three are the diseases that attract priority from sponsors, researchers, and policy makers [2, 3]. This situation is not the same as NTD, which has not gained any priority from them and even worsened [4].

The term of neglected in NTD referred to the fact that these tropical diseases are not being considered as important diseases. NTD commonly spread among poor and marginalized peoples which have limited resources [5]. NTD result in long life's deformities and handicaps, decrease productivity and economical status, and also end up many social consequences and stigmatization [6].

Though NTD is commonly found in tropical countries, it is not identical with tropical diseases. Poverty as one of NTD determinants frequently occurs in rural area, slums, and marginalized populations living nearby the equator. NTD in this area are closely associated with poverty and limited resources, such as clean drinking water access, poor sanitation, and healthy housing [7]. Poverty has reciprocal association with NTD. NTD may decrease child health, increase the health expenses and risk of ineffective treatment, decrease productivity, and result in education default [8].

NTD also contribute to most of morbidity and mortality. Women are the most vulnerable to stigmatization and discrimination after being contracted with NTD. NTD spread especially in tropical countries and particular regions. Their potential to spread in developed countries is low, because NTD are closely related with local vector and intermediate host distribution which are specifically associated with geographic region. The technology and resource to control, prevent, and eliminate NTD are available, but not for the developing countries [9].

**Figure 1:**
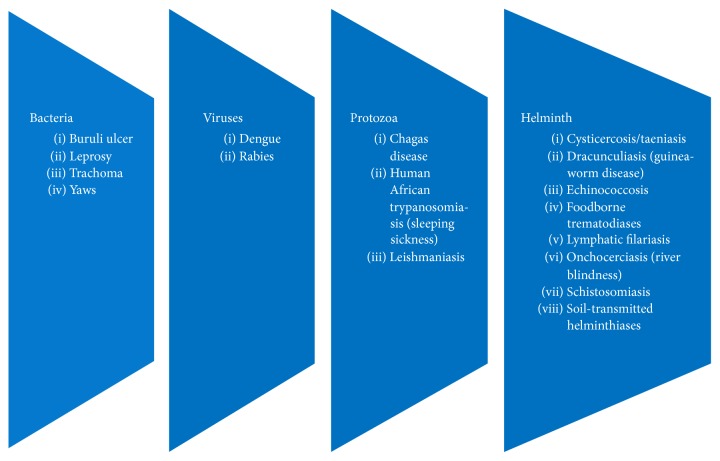
Diseases included in neglected tropical diseases (NTD) by WHO according to the causative agents.

WHO has included 17 diseases caused by bacteria, virus, and protozoa into NTD (Figure 1). The bacterial NTD are Buruli ulcer, leprosy, trachoma, and yaws. Viral NTD are dengue virus infection and rabies. Protozoa caused NTD are Chagas diseases, trypanosomiasis, and leishmaniasis. Helminths caused diseases that dominated NTD: taeniasis, dracunculiasis, echinococcosis, trematodiasis, filariasis, onchocerciasis, schistosomiasis, and soil-transmitted helminthiasis [9].

The Ministry of Health of Indonesia reported only five NTD that can be found in Indonesia, that is, leprosy, filariasis, schistosomiasis, soil-transmitted helminth, and yaws [10]. However, this review describes more than those five diseases and aims to address the magnitude of the problem of NTD in Indonesia.

## 2. Leprosy

Leprosy is an infectious disease is caused by* Mycobacterium leprae*, a road shaped bacteria classified in the same genus as* Mycobacterium tuberculosis*. Clinical manifestation of leprosy is dominated by skin and peripheral nerve disturbance. Leprosy may be complicated with deformity and handicap, which may decrease the ability of the patient to work in daily life. However, treatment by using antileprosy drug combination significantly cures and prevents disability [11].

Leprosy was a serious problem in Indonesia at 1980, with 126,221 cases in 1985. The program applied in the country decreased the prevalence to 17,539 cases in 2000. The prevalence was 86% decreasing within 15-year period. It was noted that the significant improvement in leprosy control was because of massive promotion of leprosy prevention and multidrug therapy (MDT) intervention in more than 5,600 primary health centers in Indonesia [12].

Government of Republic of Indonesia declared that leprosy had been eliminated nationally in 2000. However, the new leprosy case finding in 2000 was not significantly different with year 2013 (Table 1). Fourteen provinces and 160 districts, which mostly are located in Java island, reported prevalence of >1 per 10,000 population in 2009. Leprosy epidemical indicators were significant, such as deformity grade 2 10.5%, leprosy cases in children 12.01%, and 82.43% multibacillary (MB) cases [10, 13–15]. Indeed, intensive efforts are needed to eliminate leprosy in Indonesia.

The problems of leprosy in Indonesia are summarized as follows:

1. Early detection.
2. Development of more effective treatment.
3. Development of more effective vaccine.
4. Understanding immunopathogenesis of peripheral nerve damage.
5. Management of chronic erythema nodosum leprosum (ENL).
6. Deformity and stigmatization.

Deformity and stigmatization are serious problems among leprosy patients in Indonesia. Survey conducted in Subang district (West Java); Gresik and Malang district (East Java); Bone and Gowa district (South Sulawesi) showed that deformity in leprosy patients occurred in 76.7% of patients. Proportion of grade 2 deformity was 48.7%, while grade 1 was 28%. Almost 60% respondents were experienced handicaps during their life, including participating to social activity. At least 35.5% of the patients are experiencing stigmatization [16].

MDT for leprosy was recommended since 1982 [17]. MDT has significant impact to decrease leprosy prevalence globally. However, there are various substantial operational and technical problems among countries and territory [18]. One of the technical problems is the resistant of* M. leprae *against MDT. Resistance of* M. leprae* is a serious problem because of limited effective antimicrobes for leprosy.

Resistance against dapsone, that was applied as monotherapy, was initially reported in 1964 at Malaysia [19]. Since then, resistance to dapsone and other drugs was reported [20]. Theoretically, combination of more than two drugs with different action mechanism will reduce the probability of resistant in Mycobacteria [21]. National surveillance of* M. leprae* resistance against MDT should be established to provide accurate data. Hitherto, there is no comprehensive data available. Sporadic and partial reports were not sufficient to measure the problems and establish the programs to reduce the prevalence and spread of resistance against MDT.

Phenotypic testing is the basic method for susceptibility testing of antimicrobial agents. Even in the new proof of concept which combines the classical growth-based phenotypic test and DNA based approach, pure culture of bacteria are definitely needed for this purpose [22].* M. leprae* culture is great challenge. There is no artificial media available for* M. leprae *in vitro culture [23]. Efforts were documented to culture* M. leprae* in artificial media and animal models [24, 25]. Now, genotypic approach for susceptibility against antileprosy drugs testing was preferred. Mutations in the* folP1*,* rpoB*, and* gyrA* genes are responsible for resistance to dapsone, rifampicin, and ofloxacin, respectively. To test the susceptibility of* M. leprae *against particular antibiotics, mutations responsible for drug resistances were screened [26–28]. Screening in North Maluku and North Sulawesi showed that resistance to dapsone occurs as 0.8% in new cases and 10% in relapse cases, while rifampicin occurs with 3.3% in new cases and 20% in relapse cases [29]. There is higher prevalence of dapsone and rifampicin resistance among relapse cases comparing with new cases. It was noted that the incidence of resistance against dapsone and rifampicin in Indonesia almost did not change between the time monotherapy was introduced and after MDT was recommended by WHO [29, 30].

## 3. Yaws

Yaws is a* Treponema pallidum* subspecies pertenue infection, which may develop as chronic and recurrent disease. Yaws is nonsexually transmitted treponemal infection, which is latent and asymptomatic for years with positive serology result. Small portion (10%) of the patients will undergo bone destruction leading to deformities. Diagnosis of yaws is commonly based on the clinical findings and serology [31, 32]. Confirmatory diagnosis of yaws is using serological test, which includes nontreponemal agglutination tests, such as rapid plasma reagin (RPR) and Venereal Disease Research Laboratory (VDRL) test, and also treponemal tests such as* Treponema pallidum* hemagglutination assay (TPHA),* Treponema pallidum* particle agglutination TPPA, and fluorescent treponemal antibody-absorption (FTA-Abs). The accuracy of yaws serological test is hampered in the area which syphilis, caused by* T. pallidum* subspecies pallidum, is endemically circulated [31]. There was cross reaction between the two diseases [33].

Yaws spreads in 18 provinces of Indonesia. Five of these provinces have high burden of yaws. Yaws was reported in 68 (14%) of 497 districts in Indonesia, mostly in the eastern part of Indonesia. East Nusa Tenggara province reported 2,800 cases in 2012, which is scattered at 566 small islands in this region [32]. There were 2,112 yaws cases in 2001 and spiked to 8,907 cases in 2007. However, it gradually decreases to 5,319 cases in 2011 and 3,476 in 2012 [10, 34].

There are several obstacles to eradicate yaws in Indonesia: geographically the yaws patients are in a remote area including rural and small islands which are not easy to handle, difficulty to give benzathine penicillin for children, and also local political condition that is somehow counterproductive to the programs [34]. WHO planned to eradicate yaws globally in 2020 with adoption of Morges strategies [34]. This program intends to introduce a mass treatment in the endemic regions by using azithromycin. However, benzathine penicillin can be used as back up in case of azithromycin is not indicated, treatment failure, or in places where azithromycin is not available. This treatment followed an active survey every six months to detect and treat the remaining cases [32, 34].

The Ministry of Health launched yaws eradication program, aimed to eradicate yaws in 2013 [10], one year later than WHO target [35]. There were several approaches conducted, such as active screening new cases and contacts, empowering community, improving the capacity of health worker to diagnose and manage yaws, and intersectoral collaboration approaches [10]. Access to health service is limited in the yaws endemic area, which obstacle the passive screening program.

Munir et al. [36] reported a survey among health workers in primary health center, elementary school teachers, and parents in Muna, Southeast Sulawesi. They were asked about yaws to measure their knowledge about sign and symptoms, causative agent, and therapy for yaws. The study found that their knowledge is remarkably low. Another report from Southwest Sumba showed clean water and healthy behavior is the risk factor for yaws [37]. These reports showed that comprehensive intervention is needed to boost the community participation for yaws eradication. Reporting system for yaws incidence is very important to support the best intervention for yaws eradication. Health workers are assigned to find and report the yaws cases in the field. Learning the fact that their knowledge about yaws is remarkably low, it may diminish the validity of yaws reporting system. Insufficiency of this sector may result in delay of yaws eradication and increasing yaws contact population. Yaws eradication problem in Indonesia is summarized as follows:

1. Early detection by health workers.
2. Geographical problem of endemic area.
3. Surveillances and reporting system.
4. Inadequate confirmatory laboratory test.

## 4. Dengue

Dengue virus consists of four serotypes: DENV1, DENV2, DENV3, and DENV4. These viruses are responsible for infectious diseases with width spectrum of clinical manifestation: dengue fever (DF), dengue hemorrhagic fever (DHF), and dengue shock syndrome (DSS) [38, 39]. The four serotypes are currently circulated in Indonesia [40, 41]. Dengue virus infection is an arbovirus disease, which in Indonesia is primarily transmitted by* Aedes aegypti* and* Aedes albopictus* [42].

The incidence of DHF significantly increased since reported initially in 1968 (0.05/100.000) to 35–40/100,000 in 2013. However, in 2010, there was an extreme surge of DHF incidence to 86/100,000. The significant increasing of DHF incidence was parallel with decreasing of case fatality rate (CFR), which is 41% in 1968 to 0.73% in 2013. DHF was commonly involved in 4–15-year-old patients. However, it was noticed that from 1999 there was a trend of shifting to adults [43]. DHF incidence described here is believed not to be able to draw a clear picture of all DENV infections. DENV infection was described as pyramid with asymptomatic cases as the basis. DF, DHF, and DSS are, respectively, structured on top of it [39]. However, the proportion of those three clinical manifestations still becomes an enigma, although the awareness to the diseases, surveillance programs, diagnostic tools, and management of DENV infection in Indonesia have been significantly improved [44].

DENV is transmitted through mosquitoes' bites. It has been realized that controlling adult mosquitoes is prone to fail reducing DENV transmission. The vector control approach should aim to interrupt the transmission cycle at an early phase through immature mosquito control, which is including larvae and pupae stages. Immature mosquitoes control is believed to be more effective [45]. The vector control approach is still considered effective while vaccine and prophylaxis remedies continuously develop. There were several approaches developed and implemented for DENV vector control [46]. It seems that the socioecological approach is the best candidate to be implemented in an endemic country, such as Indonesia. Community and intersectoral approach are significantly important fundamentals of integrated public health strategies for dengue vector control [47, 48].

As dengue infection has wide spectrum of clinical manifestation, the clinical diagnosis is confusing. There are guidelines issued by WHO to respond to the need of applicable tool for clinician: WHO classification system [49] and Dengue and Control Study (DENCO) revised clinical management [50]. The study concerning the two guidelines showed that in Surabaya, Indonesia, the DENCO revised clinical management guideline superior in detection of severe dengue cases [51]. However, in a study in Semarang, Indonesia, the DENCO guideline has failed to show its superiority [52]. It is true that the DENCO guideline needs to be adjusted in terms of geographic and age-related variations issues [53]. There are many factors that may be related with clinical manifestation of dengue virus infection, including human genetics involvement which is still controversially related with population, race, and geography [54, 55].

Several methods are available for laboratory test confirmation of DENV infection: virus isolation, genome detection, antigen detection, and antibody detection. The first three methods are direct detection methods which were considered as more specific and able to confirm earlier onset of DENV infection. However, these methods are sometimes not applicable because of limited resources. The antibody detection is widely used in the field because they are easy to perform and less expensive, though it is not as powerful as direct detection especially in secondary infection [50, 56].

## 5. Rabies

Rabies is an acute, progressive, incurable viral encephalitis disease that is transmitted from animal, mainly dogs, to human. It is caused by rabies virus (RABV), a member of the family Rhabdoviridae, genus* Lyssavirus* [57]. Rabies is endemic on all continents globally. The highest incidence is concentrated in Asia and Africa. Despite the fact that rabies is 100% vaccine-preventable infectious disease, it potentially threatens over 3 billion people in the world. It is responsible for thousands of deaths every year. However, poor surveillance, underreporting, frequent misdiagnosis, and poor coordination among sectors may lead to underestimation of the burden of the disease [58].

Ten countries of ASEAN have been declared Rabies-Free ASEAN by 2020 in the occasion of The Thirty-Fourth Meeting of The ASEAN Ministers on Agriculture and Forestry (34th AMAF), on September 2012. This indicated the seriousness of the countries in Southeast Asian region to eliminate rabies.

Rabies was reported in 24 provinces out of 34 provinces in Indonesia [59]. Reported human rabies in Indonesia is small (206 cases in 2010 and significantly decreased to 119 cases in 2013) compared to thousands of cases reported in other countries such as India, China, and other Asian countries [58]. To control rabies transmission, Indonesian government uses one health approach which facilitates the multidisciplinary participation in management of zoonosis diseases.

Rabies was introduced to Bali in 2008 and progressively transmitted to the whole island [60]. The exact origin of the RABV circulating in Bali is still an enigma. However, Kalimantan and Sulawesi, other islands of Indonesia, are the most plausible hypothetical origin of the Bali RABV strains [61]. Bali was the province with the highest incidence of rabies in Indonesia. Totally there were 133 reported human deaths because of rabies in Bali from 2008 to September 2011. The highest number of reported rabid human deaths (82) was in 2010. The number of rabid human deaths was reduced to 19 during 2011. This significantly decrease of rabid human death within 3 years is a good model of rabies control for other regions or countries. It was proposed that this success is mainly because of mass vaccination program for dogs, which is the primary (98%) vector of rabies in Bali. However, the number of reported humans bitten by dogs remained over 4,000 per month. The preparedness to human rabies should be maintained, as the threat of human rabies escalation is still high [62].

## 6. Lymphatic Filariasis

Lymphatic filariasis (LF) is caused by worms inhabiting the lymphatics [63–65]. Approximately 65% of total cases are found in the Southeast Asian region [66–68]. LF cases were found in all provinces of Indonesia. In 2009, there were 11.914 LF cases reported. The number of total cases was steadily reported until 2013. There were 11.912 cases registered in 2013 [15]. LF prevalence in Indonesia varied from 0.5 to 27.6%, in which the highest rates were found in Maluku, Papua, West Papua, East Nusa Tenggara, and North Maluku provinces [65]. Three lymphatic parasites are prevalently circulating in Indonesia:* Wuchereria bancrofti*,* Brugia malayi,* and* Brugia timori *[69, 70], which are transmitted by mosquitoes of five genera—*Anopheles*,* Culex*,* Aedes*,* Mansonia*, and* Armigeres* [70]. The site of adult-worm parasitism is within the lymphatic vessels, most commonly involving the extremities and male genitals [64, 65, 70]. The disease predominantly afflicts poor people in both urban and rural areas with limited resources condition, where mosquitoes as a vector might be found in high density [64, 68].

WHO launched Global Program to Eliminate Lymphatic Filariasis (GPELF), which relies on mass drug administration (MDA) approach [68, 71]. The main goal of the program is to hamper transmission of disease between mosquitoes and human beings, mainly through mass drug distribution of diethylcarbamazine (6 mg/kg) or ivermectin (150–200 *μ*g/kg) combined with albendazole (400 mg) [68, 71]. It is recommended that all people at risk are involved in this program, since patients with asymptomatic infection may have abnormal lymphatics, and that early treatment may prevent subsequent lymphatic damage [66, 69].

The efficacy of six annual rounds of MDA was studied in Alor Island, eastern part of Indonesia. The MDA approach showed a powerful deworming campaign to decrease the LF transmission. Microfilaria rates of* B. timori* decreased significantly after MDA intervention, from 26% to 0.17%. The prevalence of filarial-specific IgG4 antibodies was significantly decreased from 80% to 6%. This data showed that MDA may be recommended for other parts of Indonesia [72].

The LF national plan has implemented MDA campaigns in 2002. However, due to financial and human resource constraints, districts often provide only partial coverage of the at-risk population within the district. In 2009, the program coverage of MDA was 66.7% [10]. An implementation research was conducted in 7 subdistricts of Papua province in Indonesia. The coverage of MDA was less than 60% in the studied area. The challenge of MDA implementation in Indonesia is not the efficacy of the drugs which were given to the community. It seems that the infrastructure is the key of the implementation, which includes the availability of transportation and physical access to the target population, reliable data bases, and competence health workers. The other challenge was to gain the trust from the community member to boost the compliance of drug administration [63]. Currently, MDA has been scaled up in a geographically scattered way to address high prevalence areas and political needs [64]. Medical professionals, donors, universities, and NGOs have played a critical role in finding new cases, assessing disease burden and supporting trainings and MDA campaigns [66].

Prevention of LF depends on mosquito vectors control, which has had limited success because development of mosquitoes resistance against insecticides [69]. Urbanization of vast areas of tropical Asia, including Indonesia, has resulted in a concomitant rise in the prevalence of both* W. bancrofti *and* B. malayi *varieties of filariasis, carried by mosquitoes that breed in nonsylvatic habitats [68, 73].

## 7. Schistosomiasis

Schistosomiasis is a chronic water-borne infection caused by trematodes* Schistosoma*, mainly found in developing countries in Africa, South America, the Caribbean, the Middle East, and Asia [69, 74, 75]. In Indonesia, schistosomiasis is caused by* Schistosoma japonicum*. It is found in three isolated areas of Central Sulawesi province, namely, Lindu, Napu, and Bada Valleys [76]. Except for* Schistosoma haematobium *that is responsible for urinary tract disease, the human schistosomes primarily affect the intestine and liver. Clinical manifestations of the disease are recognized as fever with dysenteric symptoms and loss of appetite, as well as physical growth and cognitive delay in children [69, 77, 78].

Disease prevalence fluctuated between 0.3% and 4.8% in Napu Valley and between 0.8% and 3.6% in Lindu Valley [76]. However, the prevalence of schistosomiasis in both areas tended to increase during the 2008–2011 period. The parasite transmission cycle involved domestic and wild animals as reservoir [76, 79].

In 2012, WHO approved the goal of eliminating schistosomiasis in endemic countries. It focused on improving sanitary conditions and large-scale distribution of the antiparasitic drug, praziquantel to high-risk target groups, such as school-age children, child bearing age women, and individuals involved in frequent contact with contaminated fresh water [80]. Praziquantel is well-tolerated, associated with few side effects, and has a very high therapeutic index. Moreover, a single dose praziquantel administration is usually sufficient to kill all adult worms [69, 78].

Control strategies of schistosomiasis included chemotherapy, hygiene, and sanitation improvements and agroengineering. Schistosomiasis control in Indonesia has faced many difficulties even though the endemic areas are very limited [79, 81]. The core strategy of MDA should be coupled with education to the local community, rat and snail surveillance, and support to the environmental management programs including introduction of latrines and suitable water sources [81]. In the future, there should be emphasis to understand the social dynamics and social change related to schistosomiasis, which may provide more information about concrete issues to control transmission of schistosomiasis [82]. Lack of intersectoral coordination and collaboration may have occurred, possibly leading to increase transmission and reinfection rates, and be prone to control failure.

## 8. Soil-Transmitted Helminths

Soil-transmitted helminthiasis (STH) is an infection with one or more intestinal parasitic worms: roundworms (*Ascaris lumbricoides*), whipworms (*Trichuris trichiura*), or hookworms (*Necator americanus* and* Ancylostoma duodenale*) [83]. The tropical climate of Indonesia is highly favourable to support the circulation of soil-transmitted helminths in the country.* A. duodenale *is not commonly isolated compared to* N. Americanus*.* A. duodenale *is generally found in mix infection with* N. Americanus *cases [84].

Comprehensive data of STH prevalence in Indonesia is difficult to find. There are sporadic reports regarding the prevalence of the STH which come from scattered part of the country. The prevalence of STH was reported ranging from 40 to 70% in 80s to 90s [84]. However, the estimated prevalence of STH was decreased in 2005:* Ascaris* (15.2%),* Trichuris *(12.9%), and hookworms (8.4%). It was estimated that total prevalence of any STH will be 30.5% [85]. The survey conducted by Ministry of Health in elementary schools located in 33 provinces of Indonesia showed prevalence of STH was 31.8% [86].

Eight countries in Southeast Asia carry moderate to high burden of STH. India (64%) and Indonesia (16%) together are contributing 80% of the Regional's burden [85]. Drugs used for deworming, albendazole, and mebendazole are effective and inexpensive to control STH transmission and reinfection. The MDA Programme for elimination of LF which is implemented by using combination of albendazole with ivermectin or diethylcarbamazine seems to have synergistic effect to decrease the prevalence of STH [87].

STH elimination program has been implemented by Ministry of Health of Indonesia in 1995. The program targeted preschool and elementary school age [86]. However, until this moment the country still struggles to combat STH.

In conclusion, there were efforts and programs concerning NTD that were planed and implemented in Indonesia. Some of the strategy was sufficient, but others need to be strengthened. Many factors may contribute to the rendering of NTD elimination in Indonesia, such as high population, wide range geographic of the archipelago, and limited resources. By the end of 2015 NTD is still problematic in Indonesia. A comprehensive planning is needed for sustaining effort to eradicate NTD. Multidisciplinary approach is a promising strategy to help the marginalized people.

## Figures and Tables

**Table 1:** Leprosy new cases in Indonesia 2000–2013 [13–15].

Year	New cases			*New case detection rate* per 100,000 Population
	MB	PB	Total	
2000	11,267	3,430	14,697	7.22
2003	11,956	3,594	15,549	7.29
2004	12,957	3,615	16,572	7.8
2005	15,639	4,056	19,695	8.99
2006	14,750	3,550	18,300	8.27
2007	14,083	3,643	17,726	7.84
2008	14,328	3,113	17,441	7.41
2009	14,227	3,033	17,260	7.49
2013	11,107	2,039	13,146	5.29

MB: multibacillary; PB: paucibacillary.
